# After-image formation by adaptation to dynamic color gradients

**DOI:** 10.3758/s13414-022-02570-8

**Published:** 2022-10-07

**Authors:** Marieke S. Alzeer, Kiki Houwers, Maarten van de Smagt, Stefan Van der Stigchel, Marnix Naber

**Affiliations:** Experimental Psychology, Helmholtz Institute, Faculty of Social and Behavioral Sciences, Utrecht University, Room H0.25, Heidelberglaan 1, 3584CS Utrecht, The Netherlands

**Keywords:** Adaptation, Color, After-image, After effect, CIE LAB, Dynamic adapter

## Abstract

The eye’s retinotopic exposure to an adapter typically produces an after-image. For example, an observer who fixates a red adapter on a gray background will see an illusory cyan after-image after removing the adapter. The after-image’s content, like its color or intensity, gives insight into mechanisms responsible for adaptation and processing of a specific feature. To facilitate adaptation, vision scientists traditionally present stable, unchanging adapters for prolonged durations. How adaptation affects perception when features (e.g., color) dynamically change over time is not understood. To investigate adaptation to a dynamically changing feature, participants viewed a colored patch that changed from a color to gray, following either a direct or curved path through the (roughly) equiluminant color plane of CIE LAB space. We varied the speed and curvature of color changes across trials and experiments. Results showed that dynamic adapters produce after-images, vivid enough to be reported by the majority of participants. An after-image consisted of a color complementary to the average of the adapter’s colors with a small bias towards more recent rather than initial adapter colors. The modelling of the reported after-image colors further confirmed that adaptation rapidly instigates and gradually dissipates. A second experiment replicated these results and further showed that the probability of observing an after-image diminishes only slightly when the adapter displays transient (stepwise, abrupt) color transitions. We conclude from the results that the visual system can adapt to dynamic colors, to a degree that is robust to the potential interference of transient changes in adapter content.

**Supplementary Information:**

The online version contains supplementary material available at 10.3758/s13414-022-02570-8.

## Introduction

Efficiently processing the visual environment is crucial for humans (Hyvärinen et al., 2009; MacKay & Mac Kay, 2003). Information processing is metabolically expensive, as the neuronal processing of the visual system alone takes up considerable energy (Attwell & Laughlin, 2001). It is therefore important to the visual system to maximize transmitted information while minimizing processing costs (Barlow, 1961; Laughlin, 2001). One way the visual system does this is by adaptation (Hosoya et al., 2005; Lan et al., 2012; Laughlin, 1989; Sharpee et al., 2006). Visual adaptation is a mechanism by which the sensitivity of a neuron (or neural network) adjusts its firing rate depending on the exposure duration of a stimulus (for reviews from varying perspectives, see Clifford et al., 2007; Kohn, 2007; Krekelberg et al., 2006; Rieke & Rudd, 2009; Webster, 2015): most neurons are less likely to fire as stimulus presentation time within their receptive fields increases. Adaptation further strengthens when the stimulus targets exactly the feature that a neuron or neural population “prefers,” that is right at the center of their tuning curve (Clifford, 2002; Clifford et al., 2007). Adaptation to stimuli dynamically maintains sensitivity to visual changes to accommodate for the wide range of (natural) signals that neurons, with limited response ranges, are required to encode (Brenner et al., 2000; Chander & Chichilnisky, 2001; Enroth-Cugell & Shapley, 1973; Shapley & Victor, 1978). Because the visual system mainly responds to *changes* in, for example, luminance, response saturation is prevented, and novelty detection and discriminative power are enhanced. This form of adaptation operates across the entire hierarchy of visual processing, all the way from the eye’s retinal level (e.g., Smirnakis et al., 1997) to the high-level, cortically represented perceptual level (e.g., Rhodes et al., 2003)

The advantage of compressing information through adaptation also comes with perceptual side effects in the form of illusions. For example, when observers look at a red stimulus for a considerable amount of time, adaptation gradually incurs. When the red stimulus suddenly disappears, leaving a blank (e.g., gray) screen, a cyan after-image appears (M. H. Wilson & Brocklebank, 1955), approximately complementary (i.e., with an opposite content in color space) to the adapter’s initial (primary) color (Burckhardt, 1866; Koenderink et al., 2020; Manzotti, 2017; Pridmore, 2021). Such illusions, inherent to the mismatch between the physical world and its compressed subjective representation by the visual system, nicely reveal the underlying dynamics of adaptation (Mather et al., 1998). However, perceptual effects of, for instance, color adaptation are typically studied with prolonged presentation of unchanging stimuli, mostly with the intention of maximizing adaptation effects (Gibson & Radner, 1937; Hershenson, 1989; Leopold et al., 2005; Magnussen & Johnsen, 1986; H. R. Wilson, 1997; Yeonan-Kim & Francis, 2019). In this way, neurons tuned to a specific feature are targeted consistently at peak sensitivity. However, it is currently unknown how adaptation operates and affects perception when adapters dynamically change within their feature domain (i.e., changes in hue for color or changes in direction for motion), and thus subsequently stimulate different neuronal populations with tuning curves that partially overlap but with distinct peak sensitivities. Considering that our environment is continuously changing, it is relevant to learn whether temporally dynamic stimuli change, or even disrupt or reset adaptation. The degree to which a visual change after adaptation promotes recovery from adaptation strongly depends on characteristics of both the adapter and the test stimulus (van de Grind et al., 2004). When the test stimulus shares features with the adapter stimulus, the recovery from adaptation may speed up. The interaction between sequentially presented stimuli with overlapping features, as in the case of dynamic adapters, may thus potentially hamper the emergence of after-images. Conversely, even despite such recovery processes, adaptation may also continue to build up when an adapter changes in content because tuning curves are typically broad, causing stimulations *around* rather than *at* peak sensitivity. Hypothetically speaking, rapid adaptation to a broad region of a feature dimension (e.g., both red and magenta colors) would still result in an accumulated net change across multiple neuronal populations, as predicted by the distribution shift model (Mather, 1980). In the latter case, dynamically changing adapters should evoke after-images based on, for example, the average of multiple adapter contents, predicting a quick instigation of adaptation, even during short presentations of adapter content.

The latter proposition, that dynamic adapters still promote adaptation, assumes rather fast adaptation processes. In fact, electrophysiological evidence on contrast adaptation in retinal ganglion cells and motion adaptation in the visual cortex suggests the existence of such fast adaptation processes in the order of milliseconds (Akyuz et al., 2020; Baccus & Meister, 2002; Fairhall et al., 2001; Kim & Rieke, 2001; Müller et al., 1999; Oluk et al., 2016; Priebe et al., 2002; Vautin & Berkley, 1977; Wark et al., 2007). Both perceptual and electrophysiological research suggest that brief (~25 ms) adaptation to motion or shape is enough to produce after-effects (Glasser et al., 2011; Suzuki, 2001). A rather fast chromatic adaptation process has also been reported for color contrast (Rinner & Gegenfurtner, 2000; Werner et al., 2000), but these studies used static rather than dynamic stimuli. Other studies have looked at effects of dynamic adapters (Fairchild & Reniff, 1995; Spieringhs et al., 2019) to investigate adaptation on a relatively slow time scale, with long time intervals between stimulus changes. It is important to note that the aforementioned studies have applied varying paradigms to study chromatic adaptation. For instance, one paradigm used a center-surround paradigm for slow dynamic stimuli (Fairchild & Reniff, 1995). Here, the center was kept static, and the surround changed slowly. While this provides insights into how perception of a target object changes as a function of surrounding light changes, it tells little about the history of changes of the target itself. Furthermore, paradigms that made use of static stimuli (e.g., Werner et al., 2000) can provide insights into how stimulus characteristics contribute to adaptation, but do not take into account the fact that stimuli in our environment may change dynamically over time. Most importantly, perhaps, none of these studies look at after-image content. Therefore, the following two questions remain: (i) does the visual system adapt to stimuli that change in content within a domain (i.e., its feature, not its spatial location) to a degree that leaves after-images? (ii) how does adaptation to dynamic content affect the after-image content? In the current study we employ temporal color gradients as adapters to study the effects of dynamic changes on adaptation by measuring after-image probabilities and content in two separate experiments. We chose color for three reasons: (1) color is a fundamental characteristic of vision and is especially important for identifying objects and materials (Witzel & Gegenfurtner, 2018); (2) color allows the measurement of both content and magnitude variations of after-images; and (3) short adaptation intervals with colors have, to our knowledge, not yet been investigated. We show that the visual system does indeed rapidly adapt to adapters that dynamically change color, as demonstrated through the subjective report of vivid after-images.

## Method: Experiment 1

### Participants

We recruited a total of 20 participants through online advertising and distributing flyers on campus. The majority were students of Utrecht University participating for study credits. All participants provided written informed consent and we debriefed them about the purpose of the study after the experiment. The faculty ethics assessment committee of Utrecht University’s faculty of social and behavioral sciences approved this study (#21-0533), confirming that it adheres to the set of human ethical principles of the Declaration of Helsinki.

Eight of the 20 participants indicated not having systematically seen after-images. They either guessed the color of the after-images or reported the adapter’s color rather than the (complementary) after-image color (see the *Analysis* section below for details; see Experiment 2 for solutions). These participants were therefore excluded from the analysis. The remaining 12 participants (age: *M* = 24.3 years, *SD* = 3.7; females: *N* = 6) had normal or corrected-to-normal vision and showed no signs of color blindness, as confirmed with an Ishihara color blindness (protanopic and deuteranopic) test consisting of five pictures of colored numbers.

### Procedure, stimuli, and apparatus

**Fig. 1:**
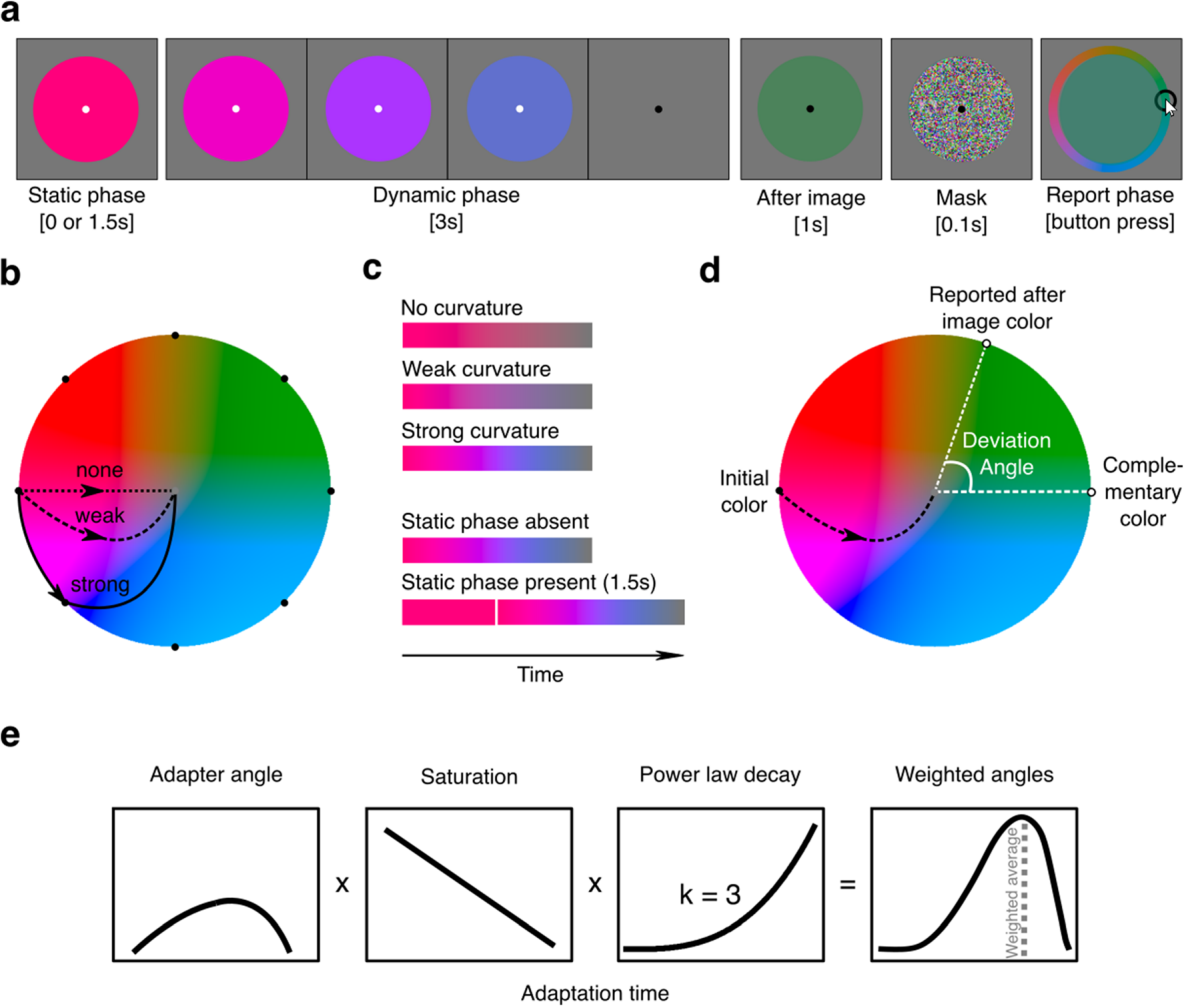
Stimulus manipulations and after-image measure – Experiment 1. **a** Stimulus presentation procedure of a single trial. **b** Color space with the adapter’s color trajectories. **c** Examples of adapter colors as a function of time. **d** Visualization of deviation angle as the dependent variable. **a–d** The initial color was chosen from one of eight locations in color space per trial (see black dots in panel **b**; equal number of trials per initial color condition). In half of the trials, the adapter statically displayed one color for 1.5 s before changing colors. During the dynamic phase, the adapter’s color gradually changed to gray, following a straight, weakly curved, or strongly curved trajectory through color space. The fixation dot turned from white to black to inform participants that an after-image should soon appear. Participants reported the after-image’s color after the presentation of a mask by clicking on a color wheel, whose center was colored depending on the position of the mouse. The dependent variable consisted of the deviation in angle between the initial color’s complementary color and the reported after-image color. **e** Modelling procedure to predict the expected after-image angle in color space (dotted line in the fourth subplot) based on the weighing of the adapter angles (first subplot) with the factors (i) saturation (second subplot), that linearly decreases over time and (ii) adaptation state (third subplot), following a power law decay over time

The experiment was programmed on a desktop computer with visual stimuli presented on a an Asus ROG swift gaming monitor (33.8 cm diagonal size; 1,920 × 1 080 pixels; 60-Hz refresh rate; maximum luminance: 300 cd/m^2^; not calibrated for anisotropies in colors and luminance). Participants sat on a chair in a darkened room with their head in a chin-forehead rest to ensure a fixed viewing distance to the screen of 48 cm. During a trial we presented a colored circle (the adapter; luminance: ~150 cd/m^2^; diameter: 8.8° visual angle) for 3 or 4.5 s (for explanation, see next paragraph), a blank screen for 1 s in which an afterimage was perceived, a mask (random colors per pixel) for 1 s to disrupt lingering after-image effects, and an interactive color wheel to report the after-image colors (Fig. 1a).

A trial started with the presentation of a gray screen with a white fixation dot (diameter: 0.2°) before the presentation of the adapter. The fixation screen was shown for a duration that was randomly chosen from a range between 0.5 and 1.5 s.

The dynamic adapter remained visible for 3 s during which it dynamically changed color (dynamic phase). In half the trials, the dynamic adapter was preceded by a static adapter for 1.5 s. The adapter displayed CIE 1976 L*a*b* (white point: d65; output: sRGB; Matlab’s lab2rgb function, using theoretical sRGB settings) colors because it approximates a perceptually (pseudo-)uniform color space and was designed to align with how human vision operates (L'Eclairage, 2004). More specifically, the orthogonal *a* (red-green) and *b* (blue-yellow) axes of color space align with the color opponency model and, at a fixed L (luminance) plane, are approximately perceptually equiluminant. The changes in colors during the dynamic phase followed a predefined color trajectory through color space, being either a straight path or a bended path from the initial color to gray, that is from full color saturation at the color space’s border (though, of course limited by the monitor’s gamut) to no saturation at the color space’s center (Fig. 1b and c). The properties of the curved trajectories were counterbalanced across trials (i.e., an equal number of trials per condition), consisting of either a clockwise or counterclockwise and either a weakly (change in angle: 0.375π radians; 67.5°) or strongly bended path (change in angle: 0.50π radians; 90.0°). In half of the trials, the adapter first remained a constant color for 1.5 s at full saturation (i.e., the static adapter phase could be present or absent). The initial start color of the adapter was randomly chosen from one of eight possible start positions (see black dots at the color space’s border in Fig. 1b; initial angles: 0π, 0.25π, 0.50π, 0.75π, 1.00π, 1.25π, 1.50π, or 1.75π radian angle in a*b color space). The fixation dot turned to black the moment that the adapter reached 0% saturation to indicate the start of the 1-s long after-image observation period.

After the after-image observation phase, participants could select the observed after-image color from a color wheel. The color wheel showed colors at 50% rather than 100% saturation to better match the saturation levels of after-images. The inner portion of the wheel changed color depending on the concentric position (i.e., angle with respect to the wheel’s center) of a computer mouse cursor. Participants clicked the left mouse button to choose an after-image color and to initiate the next trial. It was mandatory for participants to choose a color and they had to guess if they were uncertain about the color or even presence of an after-image.

Participants completed a total of 192 trials (three curvature conditions × two static phase conditions × two rotation directions × eight initial start colors × two trials per condition) and the entire experiment lasted approximately 45 min. Participants could take a break after a quarter, half, and three-quarters of all trials. To control for protanopic and deuteranopic color blindness, observers had to indicate which number they saw in five pictures taken from the Ishihara Color Vision Test before starting the experiment.

### Analysis and computational modelling

Two independent variables were of main interest, consisting of the degree of color space trajectory curvature for the dynamic adapter (*curvature type*) and the presence or absence of a static adapter phase (*static phase presence*). The dependent variable consisted of the deviation in radian angles (-π – +π) between the after-image color complementary to the initial color and the actual reported after-image color (Fig. 1d). Deviation angles of trials with clockwise trajectories were reversed (multiplied by -1) to rotate the angle in the trajectory’s direction. As such, a positive deviation angle meant that a participant observed an after-image in the extrapolated direction of the curved color space trajectory, and a negative angle, vice versa.

Participants could be removed from the analysis for two possible reasons. First, when a participant’s distribution of deviation angles was biased to (-)π rather than centered around 0 for straight-path trials, this indicated that the participant erroneously reported the adapter’s color rather than the complementary after-image color (Rayleigh test of uniformity with H1: known mean angle of 0; selection criteria: *p* < 0.001). Second, when deviation angles were distributed randomly (uniformly), without centering around a peak, this indicated that participants guessed, probably because they did not see or memorized the after-images properly (Rayleigh test of uniformity with H1: not distributed uniformly; selection criteria: *p* < 0.001).

We created a mixed linear regression model, with fixed within-subject factors of *curvature type* and *static phase presence* and a random between-subject factor predicting deviation angles. We stepwise removed insignificant main, interaction, or intercept effects. Thereafter we performed post hoc comparisons of deviation angles across conditions with paired, two-tailed Student’s t-tests.

Besides the regression model that directly linked deviation angles to single valued parameters, we created a computational model that predicted deviation angles based on the characteristics of adapter colors across time (for weighing steps, see Fig. 1e). The goal of this was not to create a model superior to models published before but to see how much variance in after-image content across conditions could be explained by a rather simple model. The model predicted deviation angles by calculating a weighed mean of the trajectory angles. Each trajectory angle was weighed by a specific integer number that was based on three parameters: (1) the duration (in milliseconds) a color was present because longer presentation times facilitate adaptation, (2) the saturation of the color because higher contrast colors facilitate adaptation (Webster & Mollon, 1994), and (3) the duration from the start of the trial following the *t*^*k*^ power law (Drew & Abbott, 2006) because adaptation to a color recovers as time passes by (for a schematic overview, see Fig. 1e). The only free parameter was *k*, optimized using a hyper-parameter grid search approach between *k* = 0--4 with a step resolution of 0.1. Note that adaptation decay may also be modeled with an exponential decay function. A power law approximates such a function. To explain how an array with weighed angles (and thus colors) was constructed, we provide several examples. An *initial* array represented the dynamic phase and consisted of 60 deviation angles per second (i.e., the screen’s refresh rate), which resulted in a total of 180 angles (3 s × 60 Hz) in the condition without a static phase. By angles we mean the relative angle between a line from the initial adapter color to the gray center and a line from the current adapters color to the gray center on the color plane (Fig. 1b). This array with angles started and ended with a 0π radian angle, but the intermediate array angles depended on the degree of curvature. For example, in the strong curvature condition, the 90th angle was 0.25π radians (45°). Another 90 times 0π radian angles (1.5 s × 60 Hz) were added in front of the array in case of the presence of a static phase, resulting in an array with 270 angles in total. These arrays only represented angles weighed for static and dynamic phase durations. To take into account effects of saturation, the initial array was extended by repeating each angle 100 (full saturation) to 0 (no saturation; gray) times depending on the color’s saturation at the specified angle and distance from the color space center. To model the release of adaptation over time, the number of repetitions for saturation was reduced by a normalized factor *f* (range: 0–1) determined with the power law function where 0 indicates fully recovered from adaptation, which applies to color angles presented at the start of the adaptation phase, and 1 indicates adaptation state at full strength, which applies to color presented at the end of the adaptation phase. For example, for the angle at 1.5 s after the start of the dynamic phase (halfway through the dynamic phase; array index = 180) with *k* set at 1.4*: f =* 180^1.4^ ÷ 270^1.4^ = 0.57. We use two examples to illustrate what numbers the final array of an adapter condition with both a static and dynamic phase constituted: (1) a static phase angle of 0π radians appeared 810 times at the start of the array (1.5 s × 60 Hz × 100% saturation × 0.09 average adaptation recovery; the latter normalization factor was based on the average of *f* of the first 90 array items). The angle of array index 180 (0.25π) was present 29 times (0.017 s × 60 Hz × 50% saturation × 0.57 adaptation recovery). The mean across all the angles in an array served as the modelled and predicted deviation angle. We computed Pearson’s correlation, mean absolute errors (MAE), and root-mean squared errors (RMSE) as indicators of model fit. The data or materials for the experiments reported here are available on request to the corresponding author. None of the experiments was preregistered.

## Results and discussion: Experiment 1

### Deviation angle results

**Fig. 2:**
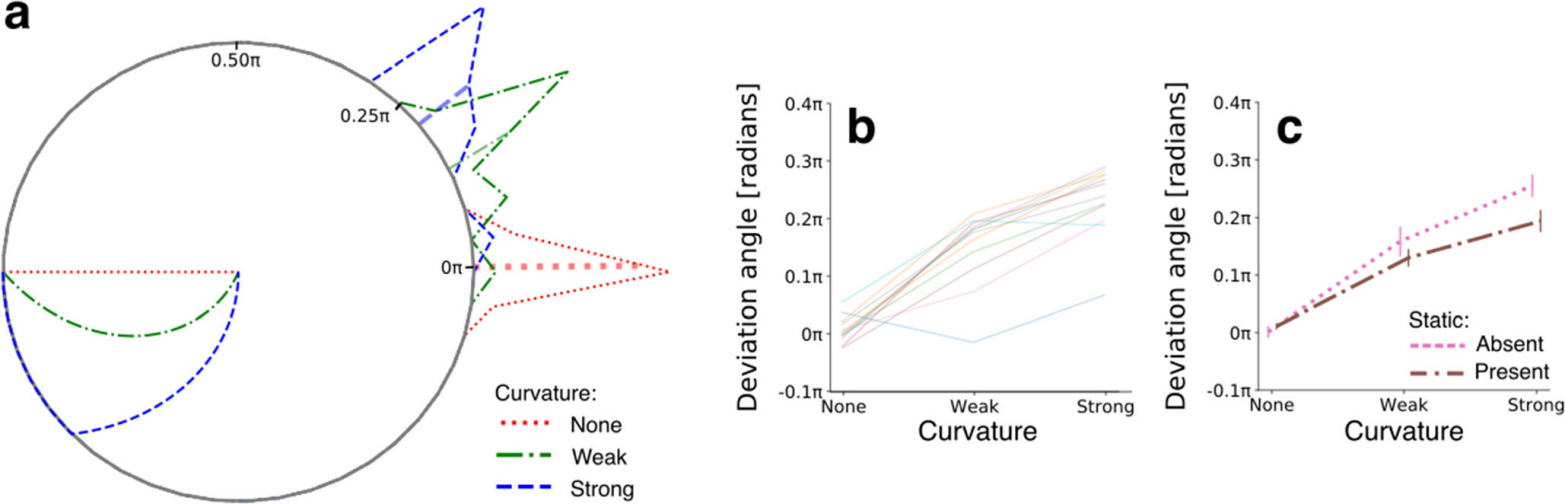
Results – Experiment 1. **a** Histogram polar plot per curvature condition (see legend) based on average deviation angle per participant, pooled across static-phase-present and -absent conditions. The radial lines extending from the circle’s border indicate the mean deviation angle across participants. Straight and curved lines within the circle represent the possible color trajectories through color space. **b** Mean deviation angle across static phase duration conditions per participant (colors). **c** Same as panel (**b**), but now the average across participants for a present or absent static adapter phase. Vertical lines display standard errors from the mean

As mentioned in the Methods, 12 out of 20 participants (60%) systematically reported having observed after-images, indicating that dynamic adapters can evoke after-images. Our next aim was to inspect the robustness of after-image colors. In other words, how variable did participants report after-image colors complementary (i.e., opposite in color space) to the initially presented colors at the start of each trial? To answer this, we calculated the mean deviation angles between (1) the colors complementary to the initial colors and (2) the reported after-image colors (Fig. 1d), per participant. We then computed histograms representing the number of participants falling within one of 45 bins distributed across the full range of 2π radians (i.e., 360°) deviation angles, per condition. Figure 2a displays these deviation angle histograms as polar plots. The red dotted lines show a distribution of mean deviation angles for the condition in which adapters went straight through color space from 100% saturation to 0% saturation, thus without changing hue. This specific distribution centers around 0π radians with a maximum deviation of approximately 0.10π radius, meaning that dynamic adapters evoke after-images of a complementary color that were robustly reported by more than half the participants.

Importantly, trials with adapters that followed a curved trajectory through color space (see green dash-dotted and blue dashed lines in Fig. 2a) evoked after-images that deviated systematically from the color complementary to the initial color, in the direction of the trajectory’s curvature (i.e., >0π radians). As shown in Fig. 2b, the patterns of deviation angles across curvature conditions indicated that the stronger the curvature, the larger the color deviation in the direction of an extrapolated color trajectory (for statistical results, see Table 1; all post hoc comparisons between curvature conditions show significant differences with *p* < 0.001). This result showed that also in the case of color the visual stimulus integrates across (color-)space and time during adaptation, leading to an integrated color after-effect.

**Table 1:** Mixed linear model for after-image colors – Experiment 1

Factor	Coef.	CI	Std. err.	z	*p*
Curvature	0.415	0.371 – 0.459	0.023	18.355	<0.001
Curvature * Static phase presence	-0.084	-0.131 – -0.037	0.024	-3.494	<0.001

For each row, numbers indicate the coefficient (weight or beta), corresponding 5–95% confidence intervals (CIs) and standard errors of coefficients, z-statistic, and p-value of significance per factor in the mixed linear model

This result is further underscored when considering the effect of a static phase preceding the curved trajectory during adaptation. The observation of a static color for 33% of the total adaptation time (i.e., 1.5 out of 4.5 s adaptation) significantly reduced the deviation angle by 27% (*SD*: 31%) in the strongly curved condition (for statistics on the interaction, see Table 1; post hoc comparison: *p* = 0.005) but did not reduce this in the straight and weakly curved condition (*p* > 0.05). The relatively weakened amount of influence of the static adapter preceding the dynamic adapter can be explained when taking the gradual recovery from adaptation into account, as is described in the following section.

### Spatiotemporal modelling results

**Fig. 3:**
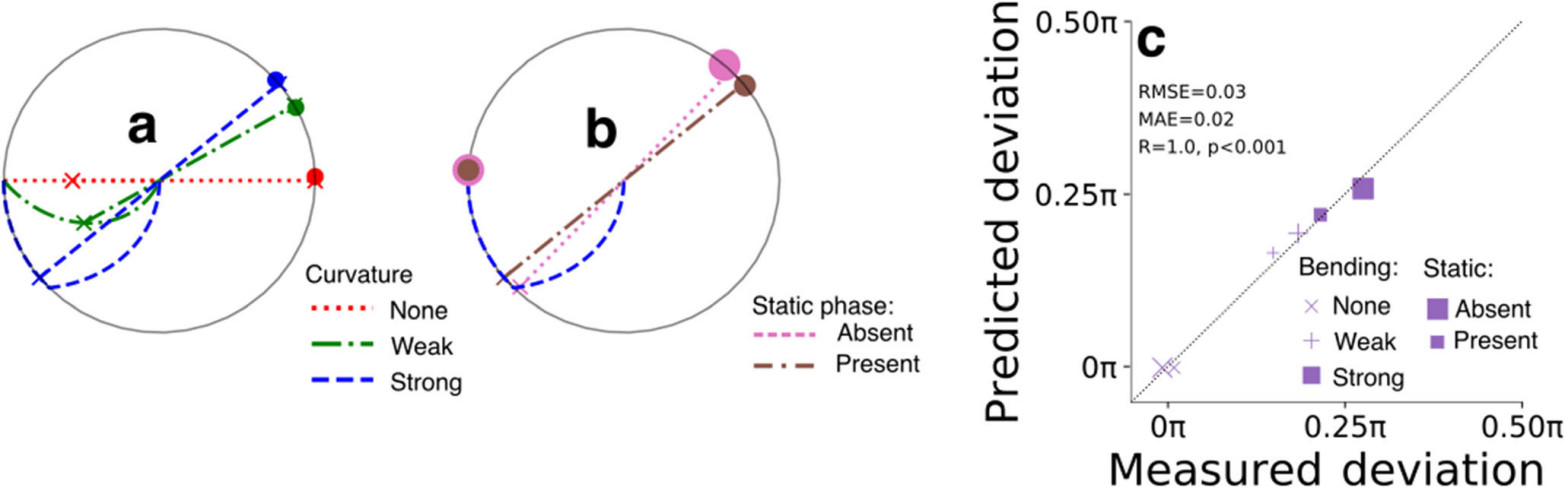
Modelling results – Experiment 1. **a** Examples of predicted deviation angles, highlighted by the straight lines from trajectories to crosses on circle border, and the measured deviation angles (median across participants’ mean deviation angle) across curvature conditions, highlighted by the dots on the circle border. **b** Same as (**a**) but now for the strongly curved trajectory for the static phase being either present (small brown dot) or absent (large pink dot). **c** Scatter plot of predicted and measured deviation angles of all possible conditions (*n* = 6). Marker symbol indicates different curvature conditions, marker size indicates static phase presence/absence. *RMSE* root mean squared error, *MAE* mean absolute error, *R* Pearson’s rho

To investigate how rapid adaptation was released as a function of time, we computationally modelled the median deviation angle per condition based on the characteristics of the adapter’s colors. Examples of the modelled angles are given in Fig. 3, showing an almost perfect fit by the model. These results indicate that a rather simple model, that bases predictions on merely three fixed, predefined adapter properties (color content, saturation, and presentation duration; see *Methods* for details) and one free adaptation release/decay parameter (a power of *k* = 1.4), very well explains the variation in after-image colors across conditions.

## Method: Experiment 2

Experiment 1 showed that (for those participants with systematic color after-effects), the color content of the evoked after-images depended on a weighted average of all presented adapter colors. However, a substantial number of participants did not observe after-images. The presence of abrupt color changes may have hampered after-image formation for these participants. We designed Experiment 2 to improve after-image formation (increasing the number of included participants) and investigate the underlying factors that determine the probability of evoking an observable after-image with dynamic adapters. To achieve this, we (1) changed the experimental design to measure and improve after-image formation probability (for examples of studies investigating after-image probability and visibility, see Atkinson, 1972; Hazenberg & van Lier, 2013; Shimojo et al., 2001), (2) varied the durations of static and dynamic phases more systematically to assess these factors in more detail, and manipulated the abruptness of color transitions to understand whether the presence of such transients affect after-image formation. More specifically, Experiment 2 differed from Experiment 1 in the following aspects: We invited a total of 55 participants. Due to COVID-19-caused restrictions, we had to change the experimental setting to an online, web-based (JavaScript) experiment. Participants were instructed to sit in front of their laptop/computer screen at a distance of approximately 50 cm. The latter was achieved by instructing participants to align the edges of two pieces of A4-sized paper behind each other, one in portrait and the other in landscape orientation, with the outer edges touching their head and screen. The participants received explicit instructions to report the color seen *after* the fixation turned black if a color was perceived at all. Participants received the additional option to indicate not having observed an after-image (press keyboard button “escape”). In addition to measuring after-image content (i.e., which color), this option allowed the measurement of the probability that an after-image was observed as a reflection of after-image magnitude (i.e., saturation/strength). We also increased the after-image phase to 1.5 s (instead of 1 s) such that participants received more time for observation, increasing the probability of observing an after-image. We presented no masks during the trials to prevent the potential disruption of after-image observations, and to partially make up for the loss of time due to the longer after-image phase.

Participants could overwrite their choice as many times as they preferred before continuing to the next trial. After their choice, participants could initiate the next trial by clicking on a “NEXT” button at the bottom of the web page.

**Fig. 4:**
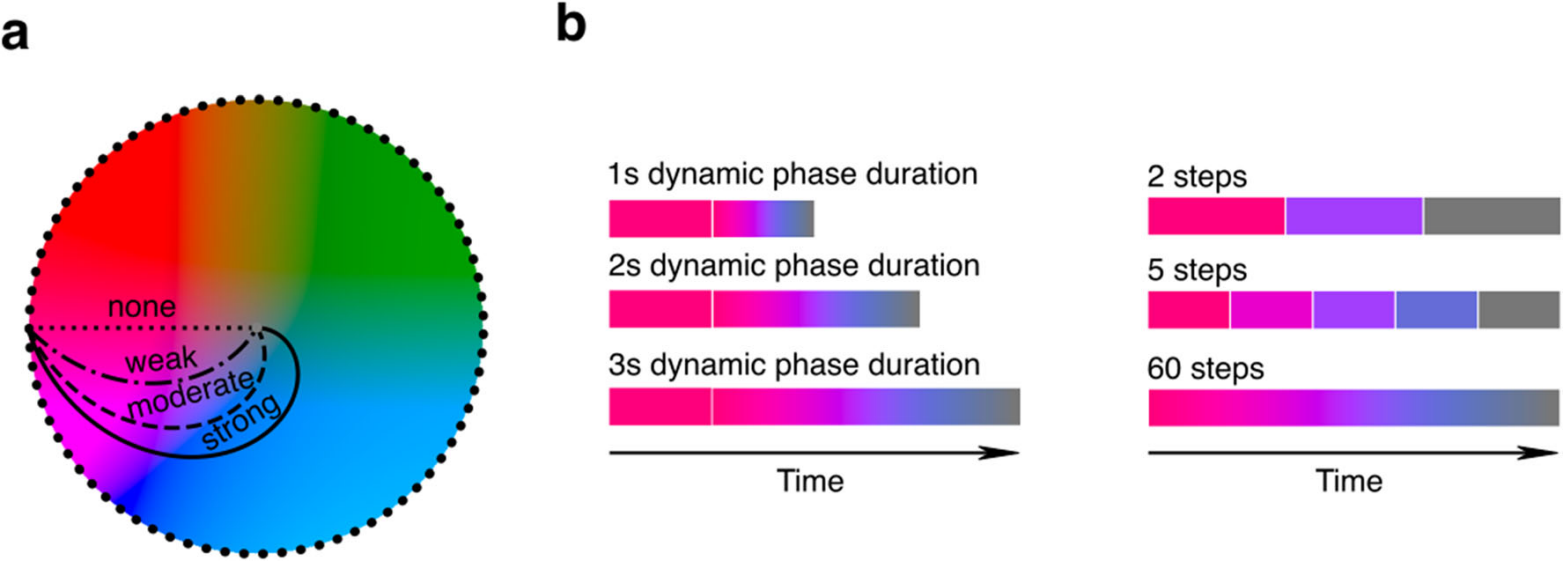
Stimuli and procedure – Experiment 2. **a** As compared to Experiment 1, the color changes of adapters in Experiment 2 followed alternative trajectories, including a stronger, swirl-like trajectory. The initial color (black dots on the color space edge) could be chosen from more options than in Experiment 1. **b** Examples of added manipulations of dynamic phase durations (1, 2, or 3 s) and number of abrupt steps (2, 5, or 60 steps/s) in color trajectories

As compared to Experiment 1, the design of Experiment 2 included modifications to the manipulations in the curvature of the adapter’s color trajectories, static phase durations, and additions of two novel stimulus manipulations to assess the generalization of the computational model attested in Experiment 1 (Fig. 4).

Firstly, we adapted the trajectories and added a swirl-like trajectory to create more datapoints for the model (Fig. 4a; change in angles: weak 0.33π; moderate 0.66π; strong 1π). Secondly, we removed the static adapter phase absence condition and varied the durations of the static phase (1 s, 2 s, or 3 s) to better model the effects of long-term adaptation. Thirdly, we manipulated the dynamic phase duration (independent of static phase duration) to model effects of short-term, fast adaptation (Fig. 4b). Fourthly, the colors changed along the trajectory in two, five, or 60 steps during the dynamic phase to examine to what degree visual transients, the smoothness of changes, and extrapolation (extrapolation should be easy for 60 steps, but more difficult for two to five steps) affects after-images. Lastly, rather than using a predefined set of eight start colors like in Experiment 1, we randomly chose an initial adapter color to prevent participants becoming acquainted with a set of colors. The experiment was programmed in JavaScript and we used the online Gorilla Experiment Builder to build and host the experiment (Anwyl-Irvine et al., 2020). Participants completed a total of 108 trials (four curvature conditions × three static phase durations × three dynamic phase durations × three step numbers × one trial per condition; rotation direction and initial start colors sampled randomly). The adapter’s and fixation diameters spanned 50% and 2% of the vertical browser size, respectively.

Eleven participants were excluded from analysis because they showed signs of color blindness according to the Ishihara color blindness test results, indicated having failed to perform the experiment in a dark room, or still did not report complementary after-image colors for straight trajectories (selection was based on same Rayleigh tests as in Experiment 1). Of the remaining population of 44 participants, 42 fell within the age range of 18–24 years and two within 25–30 years (age range instead of exact age was assessed; 27 females; 38 right-handed).

## Results and discussion: Experiment 2

### Deviation angle results

**Fig. 5:**
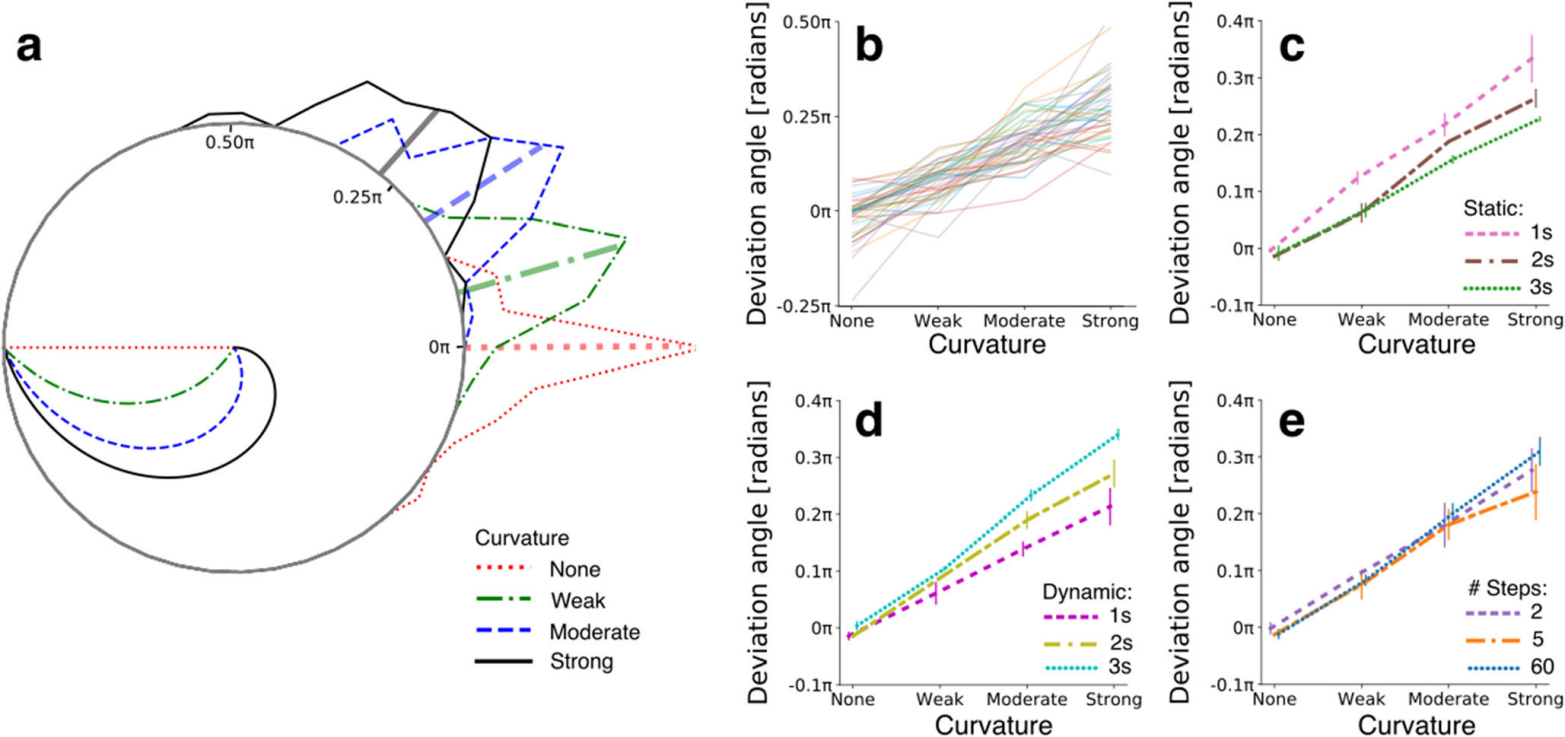
After-image color results – Experiment 2. **a**–**e** Same as Fig. 2 but now for Experiment 2 with the added manipulations of dynamic phase duration and number of steps

As outlined in the *Methods*, the majority of participants (80%) reported having systematically observed after-images with complementary colors, confirming the success of the experimental design changes implemented to increase the number of participants included in the analysis. Next, we aimed to replicate the effect of curved color space trajectories of the dynamic adapter on the after-image colors as reported previously in Experiment 1. Figure 5a and b show that all included participants robustly observed after-image colors complementary to colors that were mostly present during the dynamic phase.

The more the adapter’s trajectory curved through color space, the more likely the observed after-image color deviated from the color complementary to the initial start color (for model statistics, see Table 2; all post hoc comparisons: *p* < 0.001). Also, in line with Experiment 1, the static adapter phase duration moderated the curvature effect, with the shorter the static phase lasted, the larger the effect of curvature (Fig. 5c; all comparisons between static-phase durations for moderate curvature: *p* < 0.05; strong curvature: *p* < 0.01). Besides replicating the effects of Experiment 1, we also examined the effects of dynamic phase duration (Fig. 5d) and number of color steps (Fig. 5e). Both moderated the effects of trajectory curvature, with an enhanced effect for long as compared to short dynamic phases (comparisons between dynamic phase durations for moderate and strong curvature: *p* < 0.01) and an enhanced though small effect on strong curvature by two and 60 color steps as compared to five steps (comparisons between five steps and other step conditions for strong curvature: *p* < 0.05). The latter suggests an effect of the number of transients (an adapter with 60 color steps is perceived as a smooth change with no transients), with relatively many transients (five steps) weakening the effect of curvature.

**Table 2:** Mixed linear model for after-image colors – Experiment 2

Factor	Coef.	CI	Std. err.	z	*p*
Curvature	0.255	0.204 – 0.307	0.026	9.673	<0.001
Curvature * Static phase duration	-0.056	-0.072 – 0.040	0.008	-7.002	<0.001
Curvature * Dynamic phase duration	0.069	0.053 – 0.084	0.008	8.695	<0.001
Curvature * # Steps	<0.001	0.000 – 0.001	<0.001	2.462	0.014

For each row, numbers indicate the coefficient (weight or beta), corresponding 5–95% confidence intervals (CIs) and standard errors of coefficients, z-statistic, and p-value of significance per factor in the mixed linear model

### Spatiotemporal modelling results

**Fig. 6:**
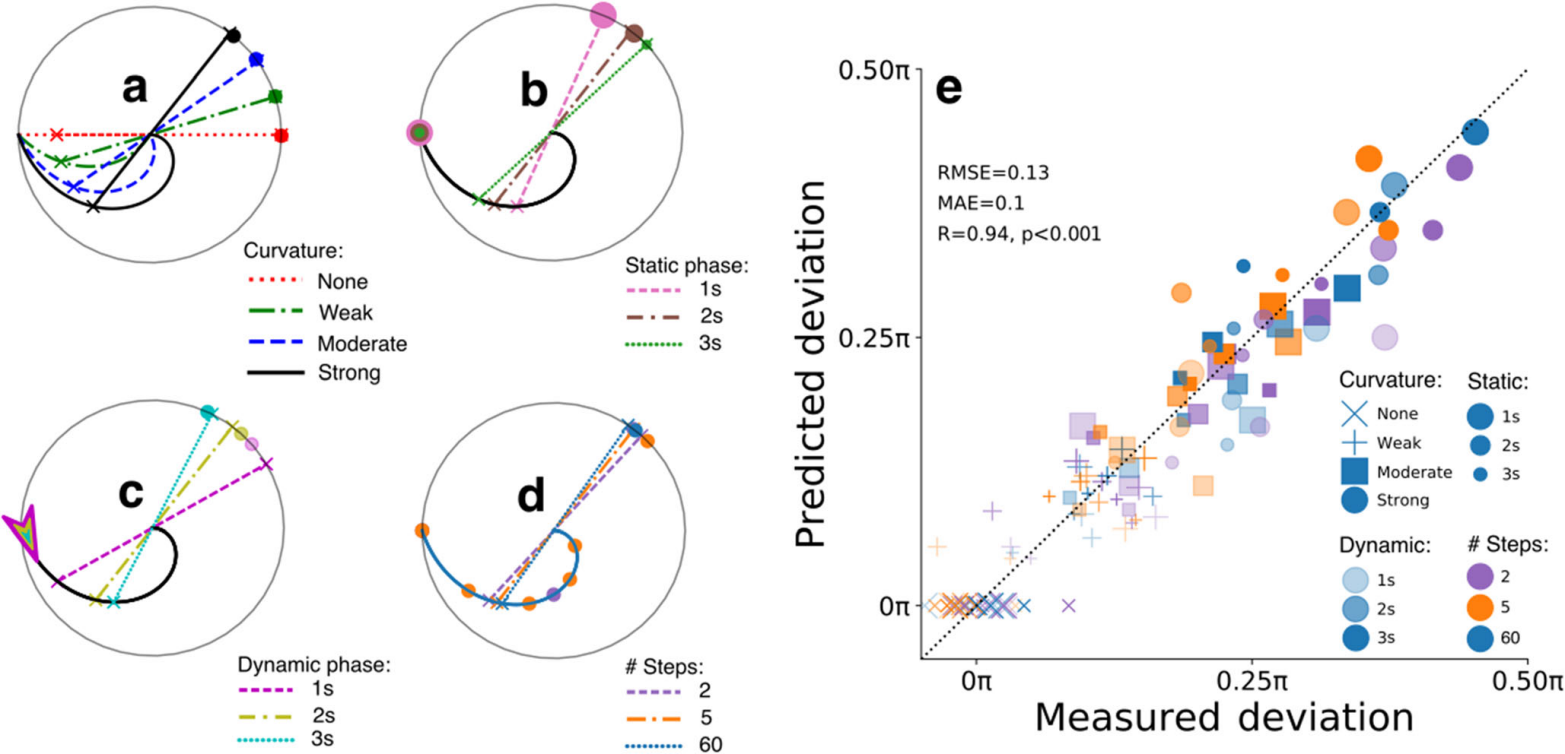
Modelling results – Experiment 2. **a**–**e** Same as Fig. 3 but now for Experiment 2 conditions (*n* = 108)

Modelled deviation angles were computed the same way as in Experiment 1. The only exception was the free parameter *k* of the power law, which was fitted at 2.0 rather than 1.4 to model a faster release of adaptation than in Experiment 1. Similar to the fit of the model of Experiment 1, the predictions matched the data (ground truth) of Experiment 2 very well (Fig. 6). The only noteworthy residual consisted of an underestimation of the effect of a relatively short (1 s) dynamic adapter phase (see magenta, dashed line in Fig. 6c and most transparent squares and circles in Fig. 6e). This undershoot of deviation angle may point at the existence of a yet unknown nonlinear process. Nonetheless, the model almost perfectly fitted the data, indicating that the model generalizes well across varying stimulus manipulations, experimental settings, and participants. Despite this achievement and the goal to create a simple model (see *Methods*), it is important to stress that much more comprehensive adaptation models exist and that the current model can be improved, for example by incorporating the non-linear relation that has been shown between where the test stimulus falls along the chromatic axis and its saturation (Webster & Mollon, 1994).

### After-image probability results

**Fig. 7:**
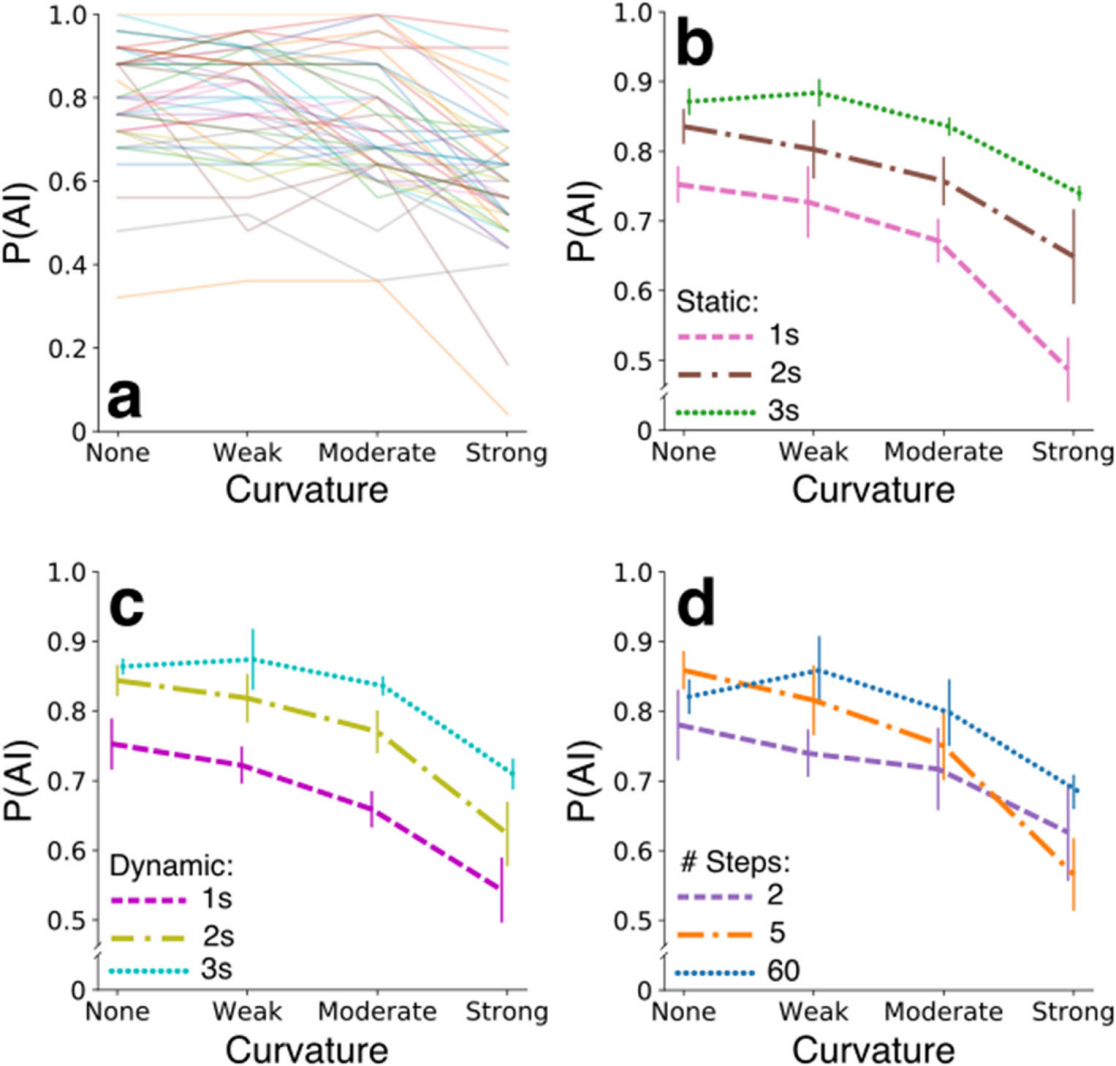
After-image probability results – Experiment 2. **a**–**d** Same as panels b–e in Fig. 5 but now for the probability of seeing an after-image

The design of Experiment 2 allowed another measurement, namely the probability that a participant observed an after-image. Figure 7 shows these probabilities across conditions. All individuals more likely observed after-images when the degree of curvature of the dynamic adapter’s trajectory through color space decreased (for model statistics, see Table 3; post hoc comparisons indicated that all curvature conditions differ significantly from each other with *p* < 0.01 except for the none versus weak condition). Long static adapter phases (comparisons across static phase durations of deviation angles averaged across curvature conditions: *p* < 0.001), long dynamic adapter phases (*p* < 0.001), and smooth color transitions (*p* < 0.05) similarly increased the probability of observing an after-image.

**Table 3:** Mixed linear model for after-image probabilities – Experiment 2

Factor	Coef.	CI	Std. err.	z	*p*
Intercept	0.522	0.466 – 0.578	0.029	18.247	<0.001
Curvature	-0.076	-0.087 – -0.064	0.006	-13.298	<0.001
Static phase duration	0.086	0.073 – 0.100	0.007	12.233	<0.001
Dynamic phase duration	0.076	0.062 – 0.090	0.007	10.715	<0.001
Curvature * # Steps	0.001	<0.001 – 0.001	<0.001	5.171	<0.001

For each row, numbers indicate the coefficient (weight or beta), corresponding 5–95% confidence intervals (CIs) and standard errors of coefficients, z-statistic, and p-value of significance per factor in the mixed linear model

When considering all directions of effects across the manipulations to adapters together, the probability of observing an after-image inversely related to the rate of change during adaptation. The more abrupt, transient color changes within a certain adaptation time window (like in the strong bending condition, 1 s lasting static phase, 1 s lasting dynamic phase, and 2 color step condition), the less likely an after-image is observed. However, what a transient actually is remains to be tested as both the curvature and the step size interact in a yet unpredicted and complex manner. A distribution shift model (as was described by Mather in 1980 for adaptation to bi-vectorial motion) may provide an alternative explanation for the pattern of probabilities across adapter manipulations. As adaptation duration increases, all color-sensitive neuronal populations, each with a peak sensitivity at a different color (i.e., with different distributions), are stimulated to reach an effective adaptation state. At the end of a curved adapter trajectory, the adapter has changed to a color that also partially stimulates and thus adapts populations that are sensitive to the opponency, after-image colors. The latter shifts the net effect of adaptation towards a more equally distributed sensitivity across color space directions, resulting in a smaller after-image formation probability.

## General discussion

We investigated how adapters that dynamically changed color produce after-images (if at all) and which adapter properties determined their content and likelihood to be observable. The analyses of results of two adaptation experiments put forward coherent evidence that dynamic adapters evoke observable after-images. Whether an adapter changes directly from one color to gray or indirectly from one color through several hues to eventually gray, any type of temporal color gradient can leave an after-image. The content of an after-image with respect to the content of a dynamic adaptor consists of a color complementary to the weighted average of the adapter’s color gradient, meaning that all and thus also shortly presented adapter colors affect the content of an after-image. The weighing depended on multiple factors of which one was assigning heavier weights to more recent (seen last) adapter colors using a power law that models a decay of adaptation as a function of time. All this points to the involvement of a rapid form of color adaptation, a finding in line with previous reports on effects of contrast, motion, shape adaptation, and color contrast (Gegenfurtner & Rieger, 2000; Glasser et al., 2011; Suzuki, 2001) in dynamic (Zaidi et al., 2012; Spieringhs et al., 2019) and static stimuli (Fairchild & Reniff, 1995; Rinner & Gegenfurtner, 2000; Werner et al., 2000). Our model is similar to the one described by Spieringhs et al. (2019); however, rapid adaptation has, to our knowledge, not been reported earlier in studies on color after-images. Furthermore, adding to the aforementioned models, we describe the relative influence of the stimulus presentation time. To model the complementary after-image colors with even better precision, we increased weights for colors with high saturation and long adaptation duration, following the classical finding that the longer and the “stronger” the adapter, the larger the effect on the after-image (Gibson & Radner, 1937; Hershenson, 1989; Leopold et al., 2005; Magnussen & Johnsen, 1986; H. R. Wilson, 1997; Yeonan-Kim & Francis, 2019). As such, our study adds knowledge to the field of chromatic adaptation from a unique perspective and with a novel paradigm.

In addition to the color content of the after-images, we investigated what determined the likelihood of observing an after-image – a binary reflection of its intensity. The probability of after-image formation apparently increases as a function of adaptation duration but decreases as a function of: (1) the number and abruptness of color changes (few and slow color changes, with long adaptation phases, enhanced after-image occurrence), or (2) the ratio of adaptation levels between initial adapter colors and complementary (opponency) colors at the end of curved color trajectories of the adapter. Nevertheless, after-images persisted to occur even for the most vivid and short-lived changes to adapters. In conclusion, adaptation does not necessarily require stable stimuli to evoke vivid after-effects. The visual system appears to adapt to a whole history of varying contents, without a strong cancellation by visual change. This means that the traditional approach to investigating adaptation with stable adapters can be extended to dynamic adapters to gain more fine-grained insights in how adaptation evolves over time with only few and short trials per participant.

The observation that dynamic adapters produce slightly weaker after-images than stable (or less dynamic) adapters requires an explanation. An adapter that rapidly changes color may trigger a mechanism that slightly hampers after-image formation. Such rapid changes are called visual transients and can affect adaptation (Naber et al., 2020), likely by operating on attentional mechanisms. More specifically, color-changing items capture attention more strongly than stable items in visual search paradigms (von Mühlenen & Conci, 2016). It is possible that the sudden appearance of a novel color may inhibit adaptation to previous colors, as a form of backward masking (Raab, 1963), and thereby suppress after-image formation. Another possibility is that color transients produce an attentional blink (Raymond et al., 1992) or enhance forward masking (Gibson, 1996), suppressing the after-image from awareness.

An alternative (neural) explanation for the observation that dynamic adapters lower the likelihood of after-image formation relates to the broadness of tuning curves of neural population sensitive to specific colors. As a dynamic adapter changes from initial colors towards complementary colors in conditions with a curved path through color space, it may also adapt populations sensitive to complementary colors through stimulation of the outer range of the tuning curves, cancelling out initial adaptation effects of opponency colors. Previous studies on motion after-effects induced a relatively complex adaptation state by presenting multiple transparent motion directions at once (Verstraten et al., 1994). Such adapters produce after-effects with a motion direction based on a weighted net effect of the separate motion components. For example, the separate adaptation to a random dot pattern with left- and rightward moving dots, results in motionless after-effects because the net adaptation level has no bias in a particular direction, similar to the here observed weakening of the color after-effect for strongly curved trajectories. While it is impossible to show two transparent colors in parallel, the relatively fast sequential presentation of multiple colors may have achieved comparable effects. Nonetheless, these propositions need to be tested in future studies. Other interesting analyses to pursue in the future are which colors are more likely to produce after-images, whether certain after-image colors deviate more strongly from the complementary color (Koenderink et al., 2020), and how the addition of luminance changes in the adapter affects after-images.

Another observation that needs to be elaborated on is the difference in the free power law parameter between the two experiments. This fitted parameter determined how fast the state of adaptation decayed, being substantially slower for the data of Experiment 1 than those of Experiment 2. Participants took part in Experiment 1 in a well-controlled lab and dark setting, likely facilitating the conditions to achieve a strong adaptation state and thus a weaker release from adaptation. On the other hand, participants sat in an environment of their choice during Experiment 2, likely with less ideal circumstances for adaptation. Another possibility is that the groups in Experiments 1 and 2 differed in adaptation recovery rates by chance as such power law parameters have been shown to be very subject dependent (e.g., see van de Grind et al., 2004).

One question that remains is where (and how) rapid color adaptation operates in the brain. The debate about the neural locus of (rapid) adaptation is currently unsettled (Barbur et al., 1999; Loomis, 1972; Shevell et al., 2008; Van Lier et al., 2009; Zaidi et al., 2012; Zeki et al., 2017) but will be of relevance to future scientific investigations that aim to better understand the underpinnings of adaptation. Another direction for future research could be to test the half-axes of the color space separately, as done before by Rinner and Gegenfurtner (2000), to get insights into interactions across adaptation in multiple, distinct color channels by looking at curves that activate both axes and to explore the mechanism behind the weighting.

## Supplementary information

ESM 1 (DOCX 456 kb)

ESM 2 (AVI 189 kb)

ESM 3 (AVI 165 kb)

## Declarations

### Submission declaration and verification

MN, MSA, KH, MVDS, and SVDS declare that this work has not been published previously, that it is not under consideration for publication elsewhere, that the publication is approved by all authors, and that, if accepted, will not be published elsewhere in the same form without the written consent of the copyright-holder.
