# Supplementary material for: After-image formation by adaptation to dynamic color gradients
Source: Atten Percept Psychophys. 2022 Oct 7;85(1):174–87. doi: 10.3758/s13414-022-02570-8 (PMC9546419; doi:10.3758/s13414-022-02570-8)
Supplement: Supplementary file 1 — (DOCX 456 kb) [file 13414_2022_2570_MOESM1_ESM.docx]

**
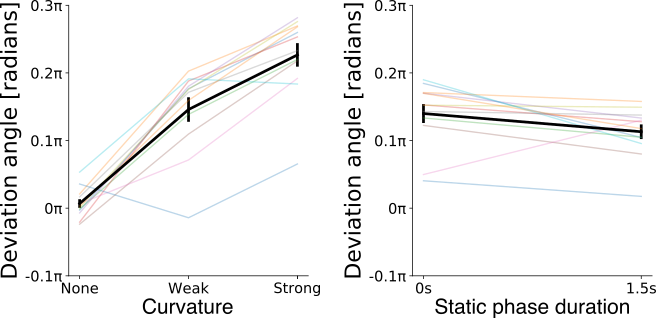
**

**Supplementary Fig.S1|Individual differences in deviation angle – Experiment 1.** Mean deviation angle per stimulus manipulation (panels) per participant (half transparent colors) and averaged across participants (black).


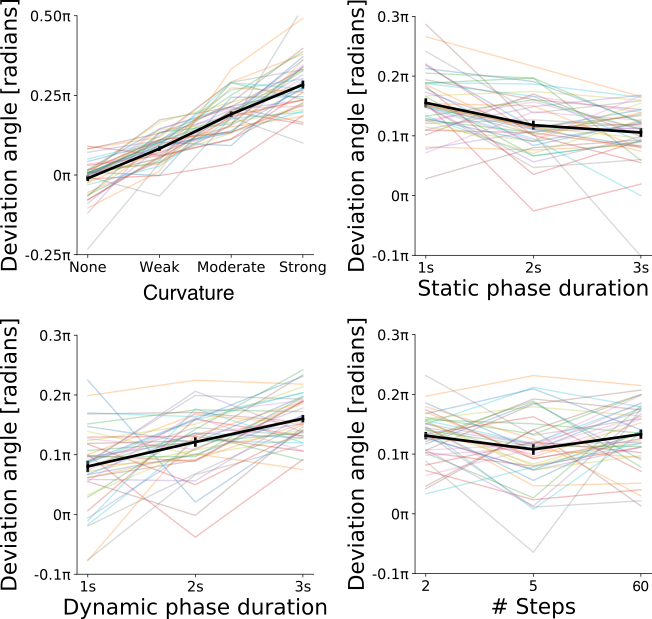


**Supplementary Fig.S2|Individual differences in deviation angle – Experiment 2.** Mean deviation angle per stimulus manipulation (panels) per participant (half transparent colors) and averaged across participants (black).


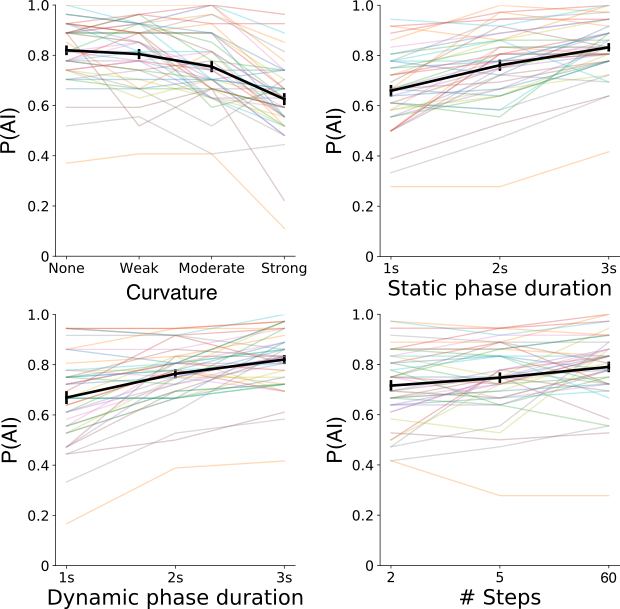


**Supplementary Fig.S3|Individual differences in after-image probabilities – Experiment 2.** Probability of seeing an after-image (P(AI)) per stimulus manipulation (panels) per participant (half transparent colors) and averaged across participants (black).
